# Comparative Evaluation of Shear Bond Strength of Various Glass Ionomer Cements to Dentin of Primary Teeth: An *in vitro* Study

**DOI:** 10.5005/jp-journals-10005-1362

**Published:** 2016-09-27

**Authors:** Rani Somani, Shipra Jaidka, Deepti J Singh, Gurleen K Sibal

**Affiliations:** 1 Head, Department of Pedodontics and Preventive Dentistry, DJ College of Dental Sciences and Research, Modinagar, Uttar Pradesh, India; 2 Professor, Department of Pedodontics and Preventive Dentistry, DJ College of Dental Sciences and Research, Modinagar, Uttar Pradesh, India; 3 Reader, Department of Pedodontics and Preventive Dentistry, DJ College of Dental Sciences and Research, Modinagar, Uttar Pradesh, India; 4 Student, Department of Pedodontics and Preventive Dentistry, DJ College of Dental Sciences and Research, Modinagar, Uttar Pradesh, India

**Keywords:** Conventional GIC, Resin-modified glass ionomer cement, Shear bond strength, Thermocycling, Type IX GIC.

## Abstract

**Aim:**

To evaluate and compare shear bond strength of various glass ionomer cements (GICs) to dentin of primary teeth.

**Materials and methods:**

Sample size taken for the study was 72 deciduous molars with intact buccal or lingual surfaces. Samples were randomly divided into three groups, i.e., groups A, B, and C and were restored with conventional type II GIC, type II light cure (LC) GIC, and type IX GIC respectively. Thermocycling was done to simulate oral conditions. After 24 hours, shear bond strength was determined using Instron Universal testing Machine at crosshead speed of 0.5 mm/ minute until fracture. Results were tabulated and statistically analyzed.

**Results:**

It was found that the shear bond strength was highest in group B (LC GIC) 9.851 ± 1.620 MPa, followed by group C (type IX GIC) 7.226 ± 0.877 MPa, and was lowest in group A (conventional GIC) 4.931 ± 0.9735 MPa.

**Conclusion:**

Light cure GIC was significantly better than type IX GIC and conventional GIC in terms of shear bond strength.

**How to cite this article:**

Somani R, Jaidka S, Singh DJ, Sibal GK. Comparative Evaluation of Shear Bond Strength of Various Glass Ionomer Cements to Dentin of Primary Teeth: An *in vitro* Study. Int J Clin Pediatr Dent 2016;9(3):192-196.

## INTRODUCTION

The human tooth is a marvel of nature. However, it has a limited capacity for regeneration. This necessitates the replacement of tooth structure lost as a result of caries, trauma, or other reasons, with a suitable restorative material.^1^ Various restorative materials have been used since years to preserve the lost tooth structure and maintain form, function, and esthetics. Dental amalgam has served as an excellent and versatile restorative material for many years. However, it has many drawbacks like lack of esthetics and the unavoidable use of mercury, which may be regarded as harmful component to the patient’s health.^2^ This leads to search more improved materials.

The glass ionomer cement (GIC) was developed with the objective to produce a restorative material that would possess the desirable properties of silicate cements and polycarboxylate cement. Conventional GICs have certain properties that make them useful as a restorative material of choice. However, some deficiencies like attack by moisture during the initial setting period, short working time, long setting and maturation time, have low fracture toughness, and exhibit lower wear resistance have limited their use to areas which are not subjected to masticatory stresses.^3^

The physical and mechanical properties of GIC were further improved when a resin portion was added to the original GIC which yielded a hybrid material, i.e., resin modified glass ionomer cement (RMGICs).^4^ It was developed to overcome the problems of moisture sensitivity and low initial mechanical strengths typical for conventional glassionomers.

Another newer generation of glass ionomer, GC Fuji IX, was developed especially for Geriatric and Pediatric patients and was introduced to clinical practice in late 1990s. It is said to possess high strength, wear resistance, a chemical adhesion to tooth structure, fluoride release, radioopacity, and less technique sensitivity to saliva.^5^ In addition, it is highly viscous, condensable, and has better esthetics. This improvement was due to reduction in the size of the glass particles in the matrix, allowing a faster speed of reaction between the silica particles and polyacrylic acid.^6^

The clinical success of restorative materials depends upon a good adhesion with dentinal surface to resist various dislodging forces acting within the oral cavity.^7^ These forces are measured in terms of compressive strength, tensile strength, and shear strength. Shear bond strength is the resistance to forces that slides restorative material past tooth structure. It assumes much importance to the restorative material clinically because of the fact that the major dislodging forces at the tooth restoration interface have shearing effect. Therefore, higher shear bond strength implies better bonding of the material to tooth.^5^

Thus, considering the importance of reliable bond strength values for restorative materials, the aim of the study undertaken is to compare and evaluate the shear bond strength of three GICs to dentin of primary teeth.

## MATERIALS AND METHODS

The materials used in the study were Dentin conditioner (GC, Tokyo, Japan), conventional Fuji II GIC (GC, Tokyo, Japan), Fuji II light cure (LC) GIC (GC, Tokyo, Japan), and Fuji IX GIC.

## COLLECTION OF SAMPLES

Seventy-two deciduous molars with intact buccal or lingual surfaces that exfoliated either due to physiologic reasons or which were indicated for extraction were collected from DJ College of Dental Sciences and Research, Modinagar. While the teeth which were rejected were the ones with caries present on both the buccal and lingual surfaces, where the crown of the tooth fractured during extraction, hypoplastic, or hypomineralized teeth or with any kind of developmental anomaly.

Debris was removed, teeth were cleaned using ultrasonic scaler, and were autoclaved. All the selected teeth were used within 3 months of collection as per recommendations of Occupational Safety and Health Administration (OSHA).

## PREPARATION OF SAMPLES

**Fig. 1:**
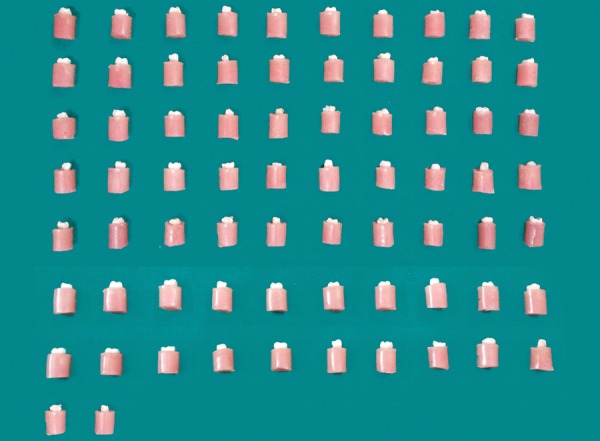
Teeth embedded in acrylic in molds

**Fig. 2:**
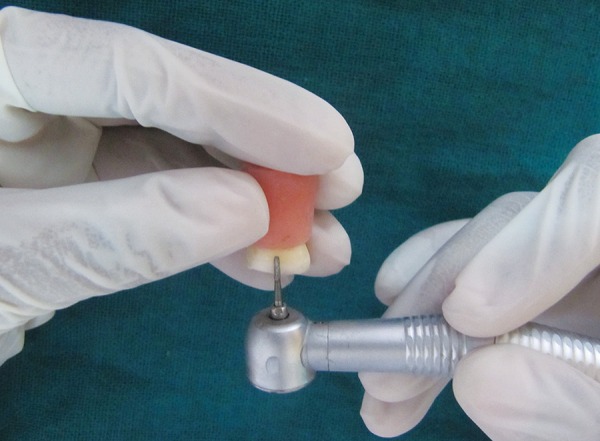
Removal of buccal/lingual surface

The specimens were embedded in acrylic resin in standardized autoclavable Teflon molds (Fig. 1). Buccal or lingual surfaces were flattened using diamond bur till yellow dentin was seen (Fig. 2). The smoothening of the flat dentinal surface was achieved with 400 number silicon carbide paper.

## RESTORATION OF SAMPLES

**Fig. 3:**
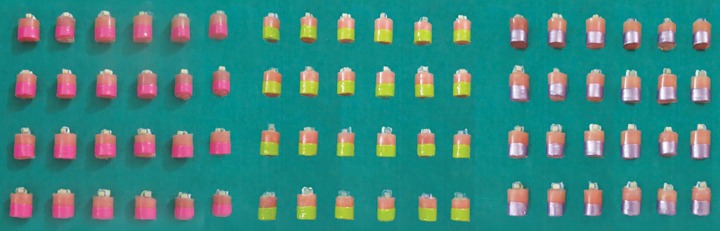
Restored samples

Prior to restoration of samples, GC dentin conditioner (i.e., 10% polyacrylic acid) was applied on all the samples for 20 seconds with a cotton-tip applicator and the samples were randomly divided into three groups and were color-coded. Thereafter the groups were restored, i.e., group A with conventional GIC (pink color), group B with type II LC GIC (green color), and group C with type IX GIC (purple color) (Fig. 3).

## EVALUATION OF SHEAR BOND STRENGTH

After restoration, specimens were stored in distilled water at room temperature for 7 to 10 days to simulate oral conditions. The dislodged specimens were rejected. A total of 12 specimens were rejected. Hence the total sample size now stands to be 60 (20 for each group).

The collected samples were subjected to thermocycling which was done in waterbaths for 500 times between 5° and 55° with a dwell time of 15 seconds in each bath and a transfer time of 10 seconds. All the teeth were kept in incubator maintained at room temperature.

**Fig. 4:**
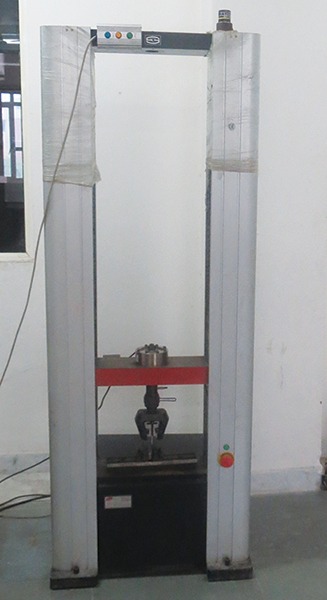
Instron universal testing machine

**Fig. 5:**
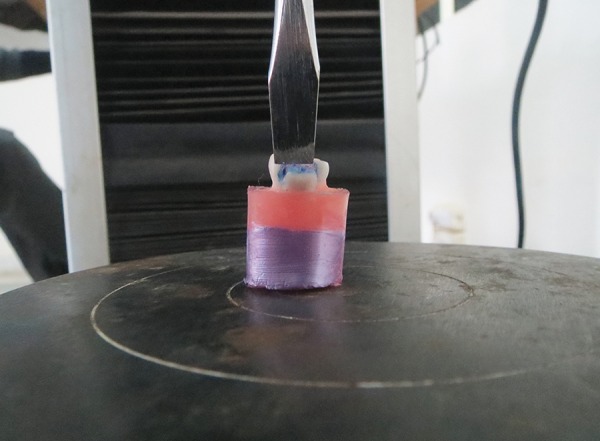
Glass-ionomer cement block dislodged

After 24 hours, the specimens were subjected to shear bond test determination using Instron universal testing machine at a crosshead speed of 0.5 mm until fracture (Fig. 4). The specimen was placed in the lower assembly of the machine and the force was applied with the help of a knife-like mandrel which engaged the GIC block and dislodged it (Fig. 5).

## Statistical Analysis

The data was statistically analyzed using independent t-test, and the intercomparison among various groups was done using one-way analysis of variance (ANOVA) and Dunnett test. Statistical significance was taken as p < 0.05.

## RESULTS

**Graph 1:**
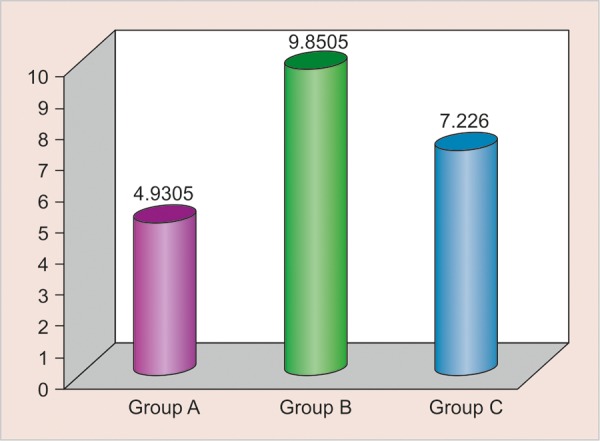
Mean values of shear bond strength of various GICs. Group A - conventional GIC; group B - light cure GIC; group C -type IX GIC

Shear bond strength was calculated according to the following formula and expressed in MPa: Stress = Failure load (N)/surface area (mm^2^). It was found that the mean value of shear bond strength was highest in group B (LC GIC) 9.851 ± 1.620 MPa, followed by group C (type IX GIC) 7.226 ± 0.877 MPa, and was lowest in group A (conventional GIC) 4.931 ± 0.9735 MPa (Graph 1, Table 1).

**Table 1:** **Table 1:** Mean values of shear bond strength of various GICs

										*95% confidence* *interval for mean*							
*Groups*		*n*		*Mean*		*Std. dev.*		*Std. error*		*Lower* *bound*		*Upper* *bound*		*Minimum*		*Maximum*	
A (conventional GIC)		20		4.931		0.9735		0.2178		4.5		5.36		2.75		6.51	
B (light cure GIC)		20		9.851		1.620		0.3624		9.14		10.56		6.68		13.01	
C (type IX GIC)		20		7.226		0.877		0.1962		6.84		7.61		5.78		9.1	

**Table 2:** **Table 2:** Independent t-test showing level of significance among various groups

*Sl. no.*		*Pair of groups*		*Probability of* *independent* *t-test*		*p-value*	
1		*Groups A & **B		0.0000		* < 0.05	
2		**Groups B & ***C		0.0000		* < 0.05	
3		*Groups A & ***C		0.0000		* < 0.05	

*A significant difference between different groups 0.05 level of significance (p< 0.05); *group A (conventional GIC); **group B (light cure GIC); ***group C (type IX GIC)

**Table 3:** **Table 3:** Distribution of variance between and within groups using one-way ANOVA test

*Source of* *variation*		*Sum of* *squares*		*Degree of* *freedom* *(df)*		*Mean* *square*		*F ratio at* *5% level of* *significance*		*p-value*	
Between groups		242.425		2		121.212		84.873		0.000	
Within groups		81.405		57		1.428					
Total		323.830		59							

**Table 4:** **Table 4:** Intercomparison of shear bond strength of various GICs

										*95% confidence interval*			
*Group (I)*		*Group (J)*		*Mean* *difference (I-J)*		*Std. error*		*Level of* *significance*		*Lower bound*		*Upper bound*	
Group A		Light cure GIC (group B)		–4.92		0.2864		0.0000*		–5.85		–3.98	
(conventional GIC)		Type IX GIC (group C)		–2.30		0.4027		0.0000*		–3.22		–1.36	
Group B		Conventional GIC (group A)		4.92		0.2864		0.0000*		3.98		5.85	
(light cure GIC)		Type IX GIC (group C)		2.63		0.4832		0.0000*		1.69		3.55	
Group C		Conventional GIC (group A)		2.30		0.4027		0.0000*		1.36		3.22	
(type IX GIC)		Light cure GIC (group B)		–2.63		0.4832		0.0000*		–3.55		–1.69	

*The mean difference is significant at the 0.05 level, i.e., p < 0.05

When intercomparison was done by applying independent t-test (Table 2), one-way ANOVA (Table 3), and Dunnett test (Table 4), shear bond strength values in all groups were found to have a significant difference at 0.05 level of significance (p < 0.05).

## DISCUSSION

The clinical success of restorative material depends upon a good adhesion with dentinal surface so as to resist various dislodging forces acting within the oral cavity.^7^ Though compressive and tensile strengths are important parameters to be evaluated but in the present study, we have evaluated shear bond strength as it assumes much importance to the restorative material clinically because of the fact that the major dislodging forces at the tooth restoration interface have shearing effect. Therefore higher shear bond strength implies better bonding of the material to tooth.^7^

In the present study, the mean value of shear bond strength was found to be highest for LC GIC, followed by type IX GIC, and was lowest for conventional GIC. All the intercomparisons between various groups were also found to be highly significant. The results are in accordance with the study done by Zafarmand and Harandi^8^ where they found that Fuji II cement was significantly better (7.4 ± 1.5 MPa) than Ariadent GICs (4.2 ± 1.9 MPa) in terms of shear bond strength. Kerby and Knobloch^9^ reported that the shear bond strength of Fuji II LC was better (11.6 MPa) as compared to conventional GIC Fuji II (6.6 MPa). In 2001, Almuammar et al^10^ found that the mean shear bond strength of Fuji II LC was more (9.55 ± 1.06 MPa) than conventional glass ionomer (3.77 ± 1.76 MPa). Torabzadeh et al^11^ noticed that the shear bond strength of Fuji II LC was highest (11.60 ± 3.19 MPa), followed by Fuji IX (6.29 ± 1.88 MPa), and the least was for conventional GIC (5.50 ± 1.94 MPa). In 2012, Nujella et al^12^ also found that RMGIC was better (9.71 MPa) than conventional GIC (3.81 MPa) in terms of shear bond strength when they did the comparison of shear bond strength of esthetic restorative materials.

A higher shear bond of RMGIC to dentin than GIC (Fuji IX GP Extra) was also reported by Poggio et al^13^ when they evaluated the effects of dentin surface treatments on shear bond strength of GICs.

The better performance of light cured GIC is due to their expected dual mechanism of adhesion or enhanced mechanical properties. The adhesion is probably through a combination of a dynamic ion exchange process and micromechanical bonding mechanism.^14^ Mathis, Ferracane^4^ considered that the enhanced mechanical properties are due to the fact that the resin acts as a reinforcing agent, resulting in significantly higher initial properties, fracture toughness during desiccation, and decreased solubility.^4^ It rapidly hardens by visible light, has shorter setting time, decreased early moisture sensitivity, extended working time, greater strength, and enhanced mechanical and physical properties.^15^

Type IX GIC is known as condensable or packable and high viscous GICs. These are characterized by having smaller glass particles and higher powder: Liquid ratio. This is said to give them higher strength, greater wear resistance, and flexural strength as compared to conventional GICs. Type IX GIC is less moisture sensitive and more resistant to dissolution than conventional GIC.^5^ Type IX GIC is different from type II LC GIC as the former has no resin in its composition, so it has a less shear bond strength as compared to type II LC GIC.

Conventional GIC is a product of an acid-base reaction between basic fluoroaluminosilicate glass powder and polycarboxylic acid.^7^ Its mechanism of bonding is based on bond formation between carboxyl groups of polyacrylic acid with hydroxyapatite at the tooth surface.^16^ The lowest shear bond strength was observed for this group. It could be because they are susceptible to attack by moisture during the initial setting period. They have short working time, long setting and maturation time. Furthermore, they are susceptible to fracture and exhibit low wear resistance.^3^ They have inferior mechanical properties like low fracture toughness, tensile strength, and brittleness as compared to LC and Fuji type IX glass ionomers. So, they are best avoided at stress bearing areas.

Based on the reported benefits of improved strengths of Fuji II LC GIC, along with the conclusions drawn from the present *in vitro* study, it can be fairly said that Fuji II LC GIC can be effectively used in the areas of stress. It holds a place in the application of posterior restorations, in minimal invasive techniques as well as for general clinical utility in pediatric dentistry.

## CONCLUSION

The following conclusions were drawn from the study:

- All the restorative materials used in the study showed significant values of shear bond strength.
- The mean value of shear bond strength was found to be highest for LC GIC, followed by type IX GIC, and was lowest for conventional GIC.
